# Investigation of Occult Hepatitis B Virus Infection in Anti-HBc Positive Patients from a Liver Clinic

**DOI:** 10.1371/journal.pone.0117275

**Published:** 2015-03-12

**Authors:** Maria Carmela Martinez, Chee Choy Kok, Cristina Baleriola, Peter Robertson, William D. Rawlinson

**Affiliations:** 1 Virology Division, Prince of Wales Hospital, Sydney, Australia; 2 SEALS Microbiology, Prince of Wales Hospital, Sydney, Australia; 3 School of Medical Sciences, University of New South Wales, Sydney, Australia; University of Pisa, ITALY

## Abstract

Occult hepatitis B infection (OBI) is manifested by presence of very low levels (<200IU/mL) of Hepatitis B viral DNA (HBV DNA) in the blood and the liver while exhibiting undetectable HBV surface antigen (HBsAg). The molecular mechanisms underlying this occurrence are still not completely understood. This study investigated the prevalence of OBI in a high-risk Australian population and compared the HBV S gene sequences of our cohort with reference sequences. Serum from HBV DNA positive, HBsAg negative, and hepatitis B core antibody (anti-HBc) positive patients (study cohort) were obtained from samples tested at SEALS Serology Laboratory using the Abbott Architect, as part of screening and diagnostic testing. From a total of 228,108 samples reviewed, 1,451 patients were tested for all three OBI markers. Only 10 patients (0.69%) out of the 1,451 patients were found to fit the selection criteria for OBI. Sequence analysis of the HBV S gene from 5 suspected OBI infected patients showed increased sequence variability in the ‘a’ epitope of the major hydrophilic region compared to reference sequences. In addition, a total of eight consistent nucleotide substitutions resulting in seven amino acid changes were observed, and three patients had truncated S gene sequence. These mutations appeared to be stable and may result in alterations in HBsAg conformation. These may negatively impact the affinity of hepatitis B surface antibody (anti-HBs) and may explain the false negative results in serological HBV diagnosis. These changes may also enable the virus to persist in the liver by evading immune surveillance. Further studies on a bigger cohort are required to determine whether these amino acid variations have been acquired in the process of immune escape and serve as markers of OBI.

## Introduction

Hepatitis B virus (HBV) is the most common transplant and transfusion-transmitted disease and the leading cause of liver cirrhosis and hepatocellular carcinoma globally. Despite the availability of an effective vaccine, the virus still infects over 350 million people worldwide, and is endemic in countries such as China. Currently both diagnosis and antiviral therapy for HBV infection primarily targets the hepatitis B surface antigen (HBsAg). The “a” epitope within the major hydrophilic region (MHR) has been identified as the dominant target of neutralising antibody. Mutations in this region can cause reduced binding of anti-HBs antibody, leading to immune escape of the virus, and continuing replication [1].

Occult Hepatitis B infection (OBI) is defined by the absence of HBsAg despite the presence of HBV DNA in the liver, blood serum, or peripheral blood mononuclear cells (PBMCs), irrespective of the presence of other hepatitis B viral antibodies and antigens[2]. OBI poses a significant risk to those receiving blood transfusions or tissue transplants because conventional donor screening with HBsAg and anti-HBc may yield serologically negative results despite the presence of HBV DNA[3]. It has been shown that 20% of OBI infections are negative for all HBV serological markers while HBV DNA is present[4] although the HBV viral load is often low[2]. This means detection of OBI can be challenging due to the extremely low levels of viral DNA (<200IU/mL) in infected individuals without detectable HBsAg[5]. At times, anti-HBc can be used as a less than ideal surrogate marker for identifying potential seropositive OBI[5].

Despite numerous studies of OBI, its prevalence is unclear. This is due to the varying prevalence of OBI in cohort studies, small sample sizes, a lack of appropriate controls, and varying assay sensitivities used in detection across testing centres[6–8]. Although there have been studies aiming to identify OBI prevalence in some populations, to date, there has been no study characterising the prevalence of OBI cases in Australia. The underlying immunological and molecular mechanisms of OBI are not completely understood. The failure of assays to detect HBsAg in OBI infection is attributable to mutations of the S and pre-S1/2 genes which may cause modifications to hepatitis B surface antigenicity[9,10]. Mutations of the S gene may be responsible for negative results in individuals tested with standard assays[11,12]. The exact mechanism by which HBsAg remains undetected is poorly understood, due partly to difficulties of full-length DNA sequencing with low levels of viral DNA typical in the sera of OBI infected individuals.

This study aimed to detect the prevalence of OBI in a high-risk cohort and investigate the mutations of HBV DNA among these OBI infected patients.

## Materials and Methods

### Sample selection

The project was approved by the Human Research Ethics Committee at NSW Health, South Eastern Sydney Local Health District, submission code AU/61177915. The samples were de-identified by a person independent of the study using a coding system blinded to the main operator. We accessed the SEALS database for serum samples that were tested for HBV markers from 2007 until 2014. Serum sample volumes ranged from 0.5–1.5mL. The ethics committee approved the consent procedure and the study was deemed to be of low/negligible risk and compliant with the Human Tissue Act, 2007. The HREC approved the right to waive procurement of consent from patients whose samples were used for this study. From the pool of HBV related tests, samples that were HBV DNA positive, anti-HBc positive, and HBsAg negative were selected for the study cohort. Testing for HBsAg and anti-HBc was performed using the Abbott Architect HBsAg Qualitative II and Abbott Architect Anti-HBc kits respectively (Abbott Diagnostics), whilst preliminary testing of HBV DNA was performed using the COBAS Ampliprep/COBAS TaqMan HBV Test v.2 (Roche Diagnostics) (LOD = 9 IU/mL). Patient consents were obtained by the physicians requesting testing for HBV, there was no access to patient records apart from HBV related tests, and these were undertaken as part of a quality exercise in the laboratory.

### HBV DNA extraction and nested polymerase chain reaction (PCR)

All samples were further tested for HBV DNA. Serum samples were concentrated using a high-speed centrifuge at 24,000*g* for 57 minutes at 4°C and DNA was extracted from 200μL of concentrated serum using QIAamp DNA Mini Kit (Venlo, Limburg, Netherlands) with a final elution volume of 50μL.

A nested PCR protocol[13] was modified in this study and used in PCR with a final amplicon size of ~1Kb that covers the HBsAg region as well as domains A to E of the overlapping polymerase region. dH_2_O was used as negative control to avoid false positives. In the first round PCR, master mix consisted of: 1x PCR Buffer (Bioline, London, UK), 2.5mM MgCl_2_ (Bioline, London, UK), 0.2mM dNTPs (Promega, Madison, WI, USA), 0.2μM forward primer 5’-AGC CCT CAG GCT CAG GGC ATA-3’ (Sigma-Aldrich, St. Louis, MO, USA), 0.2μM reverse primer 5’-CGT TGC CKD GCA ACS GGG TAA AGG-3’, 0.1μL *Taq* Polymerase per 25μL total volume (Bioline, London, UK), and dH_2_O. Amplification was performed using the following conditions: 95°C for 5 mins; 34 cycles of 94°C for 30 secs, 52°C for 30 secs, 72°C for 1 min; and final incubation at 72°C for 2 mins.

Master mix in the second round PCR consisted of: 1x PCR Buffer, 1.5mM MgCl_2_, 0.2mM dNTPs, 0.2μM forward primer 5’-TCA TCC TCA GGC CAT GCA GT-3’, 0.2μM reverse primer 5’-GAC ACA CTT TCC AAT CAA TNG G-3’, 0.2μL *Taq* Polymerase per 50μL total volume, and dH_2_O. PCR was carried out using the following conditions: 95°C for 5 mins; 34 cycles of 94°C for 30 secs, 50°C for 30 secs, 72°C for 1 min; and final incubation at 72°C for 7 mins. The amplicons were subjected to electrophoresis and confirmed by comparison to reference bands on a 1000bp EasyLadder II (Bioline, London, UK).

### PCR purification and sequencing

Purification of second round PCR amplicons was performed using Wizard SV Gel and PCR Clean-Up System (Promega, Madison, WI, USA) and polyethylene glycol (PEG) precipitation method. Purified amplicons were then used in sequencing reactions with forward primers 5’-TCA TCC TCA GGC CAT GCA GT-3’ and 5’-ACT GAG CCA GGA GAA AGC GAC TGA GGC-3’, and reverse primers 5’-GAC ACA CTT TCC AAT CAA TNG G-3’ and 5’-TAT CAA GGA ATT CTG CCC GTT TGT CCT-3’ respectively. The sequencing PCR was performed for 25 cycles at 96°C for 10 secs, 50°C for 5 secs, and 60°C for 4 mins. The sequencing products were then sent to the Ramaciotti Centre (BABS, UNSW, Sydney, Australia) for DNA Sanger sequencing.

### Phylogenetic Analysis

Four local non-occult samples sourced from SEALS at POWH dating from January 2007 until March 2013 were sequenced and used as local references. These local references had a serological profile of HBsAg positive, anti-HBc positive and HBV DNA positive. Furthermore, HBV genotype C sequences were retrieved from GenBank as references and a consensus sequence was made from seven GenBank references. All sequences were aligned using ClustalW2. Phylogenetic and molecular evolutionary analyses were conducted using MEGA version 5.2. Genetic distances were estimated using the Kimura two-parameter method, and standard errors of distances were computed using a bootstrap method (1,000 replicates). A phylogenetic tree was constructed with the neighbour-joining method, and its statistical significance was tested by the bootstrap method (1,000 replicates).

## Results

**Fig 1:**
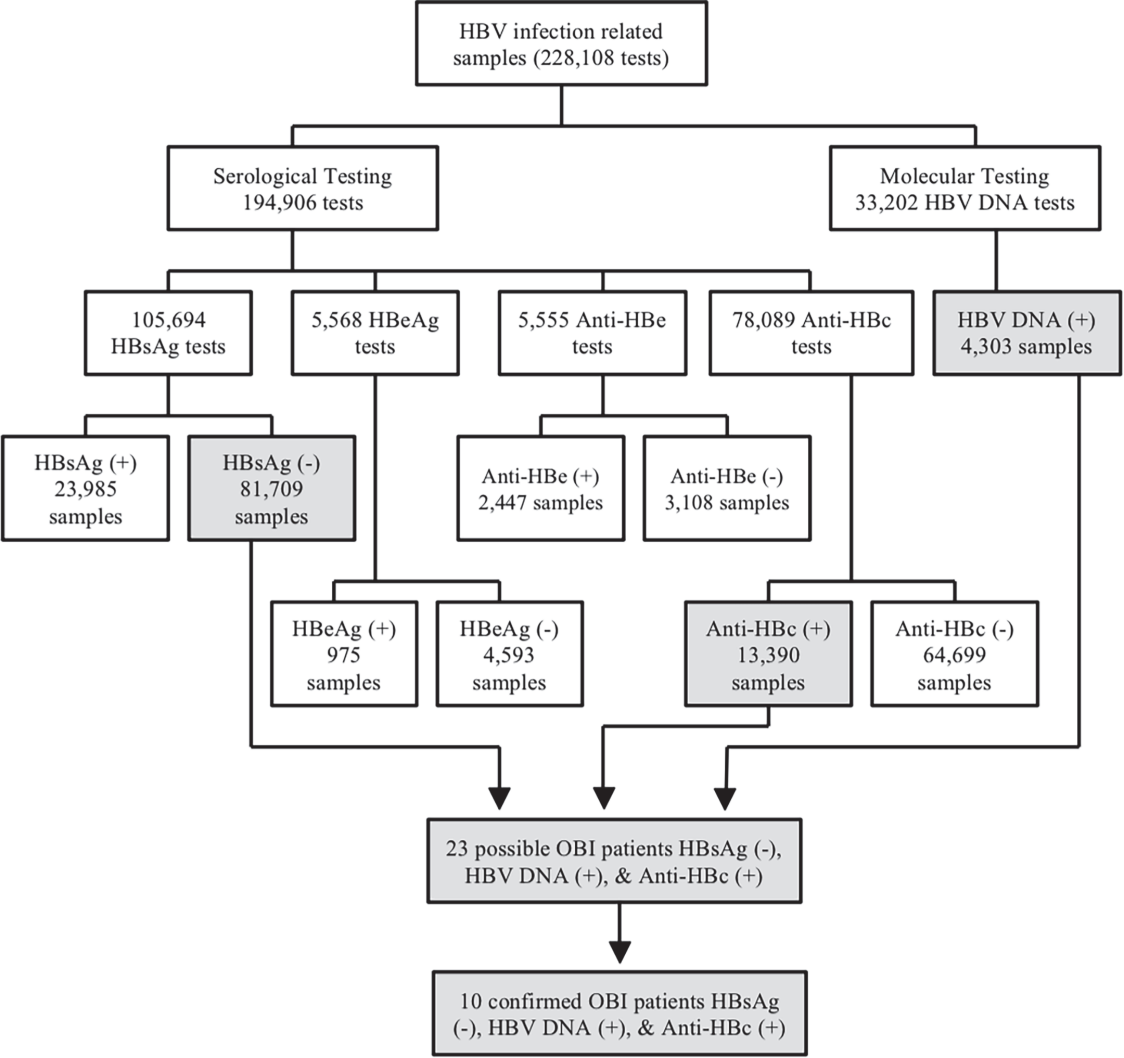
HBV infection related tests conducted by SEALS from the January 1^st^ 2007 – March 15^th^ 2013. From January 2007 until March 2013, SEALS at POWH conducted 228, 108 tests that were related to HBV infection. Of the 105, 694 samples tested of HBsAg, 81,709 were negative while 21,985 tested positive. There were 5,568 HBeAg samples tested. Nine hundred seventy-five samples were HBeAg positive while 4,593 were HBeAg negative. Additionally, out of the 5,555 anti-HBe tests, 2,447 samples were positive and 3,108 were negative. Of the 78,089 samples tested for anti-HBc, 64,699 were negative while 13,390 samples were positive. Lastly, 4,303 out of 33,202 samples tested for HBV DNA were positive. From these tests, 23 patients were possible OBI. However, only 10 patients were in compliance with the study criteria (HBsAg negativity, HBV DNA and anti-HBc positivity) that comprised out study cohort.

Samples used in the current study were sourced from an archive of all tests relating to HBV infection in the period 2007 to 2013. In the given time frame, SEALS at POWH conducted a total of 228,108 serological and molecular tests that were related to HBV infection. Of the 105,694 samples tested for HBsAg, 81,709 tested negative and 21,985 were positive. Additionally, of the 78,089 samples tested for anti-HBc, 64,699 were negative while 13,390 samples were positive for the antibody. For molecular testing, HBV DNA detection was conducted in 33,202 samples resulting in 4,303 HBV DNA positive samples. Results of other serological markers tested are presented in Fig. 1. In some cases multiple samples from the same patient were tested at different times.

A total of 1,451 patients were tested for our three OBI markers. Twenty three patients out of 1,451 (1.59%) had concurrent HBsAg negativity, anti-HBc positivity and HBV DNA positivity. However, in 11/23 patients, we were unable to confirm persistent (>6 months) low levels of HBV DNA (<150 IU/mL) and HBsAg negativity. A further two patients had insufficient and inconsistent serological data and were therefore disqualified from the study cohort. After stringent identification of sample data and availability that fit the selection criteria, a total of 10 out of 1,451 (0.69%) possible OBI cases comprised the study cohort.

**Fig 2:**
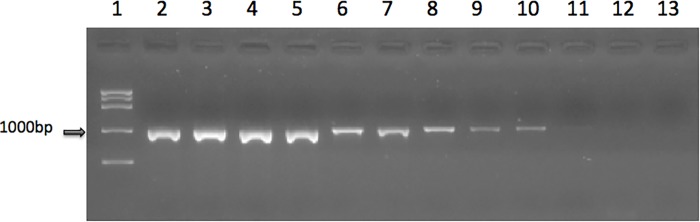
Limit of detection of designed PCR in amplifying the S gene of HBV DNA. The PCR was performed on serially diluted World Health Organization International Standard for Hepatitis B virus DNA with an initial viral concentration of 1x10^6^ IU/mL. Lane 1: 1000bp ladder; lane 2: DNA from HBV infected sample (positive control); lane 3: 20,000IU/mL, lane 4: 2,000IU/mL; lane 5: 200IU/mL, lane 6: 100IU/mL; lane 7: 50IU/mL; lane 8: 25IU/mL; lane 9: 12.5IU/mL; lane 10: 6.25 IU/mL; lane 11: 3.125IU/mL; lane 12: 1.5625IU/mL; lane 13: dH_2_O (negative control).

A nested PCR protocol obtained from Health Protection Agency[13] was employed to detect the presence of HBV viral DNA in serum. Modification of the protocol achieved a limit of detection of 6.25 IU/mL (1.25 IU/reaction) (Fig. 2). Serum from patients was subjected to viral DNA extraction and PCR. Of the 10 possible OBI cases, five showed the presence of HBV DNA in serum. The clinical and serological data are described in Table 1. Two of the five patients were female, the patient age ranged from 47–76 years, and there were 2–9 samples from each patient. Samples that showed a distinct band at 1000bp were considered HBV PCR positive and are shown in Table 1. Consequently, samples that did not show a band as a mark of successful amplification were marked HBV PCR negative.

**Table 1:** Patient List with related serological and molecular test results.

**Patient 1**	**Date of Birth: 13-09-1945**		Sex: Female					
Sample Name	Date Sample Collected	HBsAg	anti-HBc	HBeAg	anti-HBe	anti-HBs (IU/L)	HBV DNA (IU/mL)	HBV PCR
1A	26/08/2011	-	n/a	-	+	2.3	not detected	+
1B	30/05/2008	-	n/a	n/a	n/a	n/a	not detected	-
1C	24/12/2007	-	+	-	-	n/a	<12	+
1D	13/09/2006	+	+	-	-	n/a	n/a	+
**Patient 2**	**Bate of Birth: 25-02-49**		**Sex: Male**					
Sample Name	Date Sample Collected	HBsAg	anti-HBc	HBeAg	anti-HBe	anti-HBs (IU/L)	HBV DNA (IU/mL)	HBV PCR
2A	14/02/2008	-	+	-	n/a	n/a	<12	-
2B	19/09/2006	-	n/a	n/a	n/a	n/a	n/a	+
2C	24/01/2005	-	n/a	n/a	+	n/a	n/a	+
**Patient 3**	**Date of Birth: 13-01-56**		Sex: Male					
Sample Name	Date Sample Collected	HBsAg	anti-HBc	HBeAg	anti-HBe	anti-HBs (IU/L)	HBV DNA (IU/mL)	HBV PCR
3A	23/09/2008	-	+	-	+	39.34	<12	+
3B	18/02/2008	-	+	-	+	0	<12	+
3C	13/10/2006	+	+	-	+	1.32	133	+
Patient 4	Date of Birth: 2-11-33		Sex: Female					
Sample Name	Date Sample Collected	HBsAg	anti-HBc	HBeAg	anti-HBe	anti-HBs (IU/L)	HBV DNA (IU/mL)	HBV PCR
4A	4/04/2013	-	+	n/a	n/a	87.56	n/a	+
4B	4/10/2012	-	n/a	n/a	n/a	n/a	n/a	-
4C	5/01/2012	n/a	n/a	n/a	n/a	52.14	n/a	-
4D	6/10/2011	-	+	n/a	n/a	52.02	n/a	-
4E	7/07/2011	-	n/a	n/a	n/a	n/a	n/a	-
4F	6/04/2011	-	n/a	n/a	n/a	n/a	n/a	-
4G	7/10/2010	-	+	-	-	14.12	n/a	-
4H	5/10/2010	-	+	n/a	n/a	13.5	n/a	-
4I	29/09/2010	-	+	-	-	n/a	<20	-
Patient 5	Date of Birth: 19-11-61		Sex: Male					
Sample Name	Date Sample Collected	HBV sAg	HBV cAb	HBV eAg	HBV eAb	HBV sAb (IU/L)	HBV DNA (IU/mL)	HBV PCR
5A	10/10/2012	-	+	-	-	n/a	31	+
5B	24/06/2009	-	+	n/a	n/a	n/a	n/a	+

The amplified 1000bp fragment encompassing the S gene was purified and sequenced. Initial comparison with reference sequences from GenBank revealed that all five patients in our cohort carried sequences resembling the HBV genotype C (data not shown).

**Fig 3:**
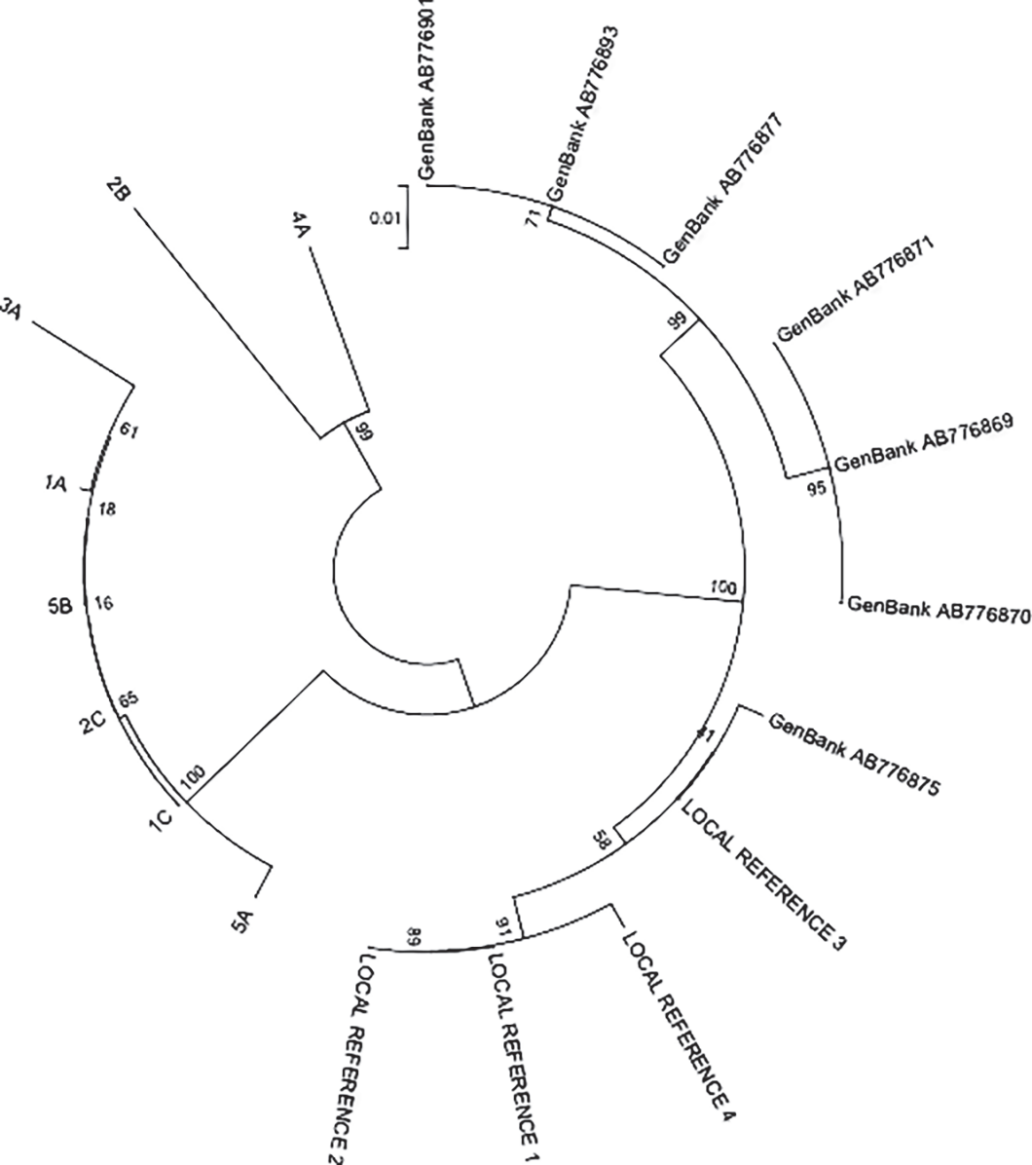
Cluster sequence of OBIs, non-OBI local references and GenBank sequences. An unrooted phylogenetic tree of HBV DNA S gene was constructed using the neighbour-joining method using eight OBI sequences, four local non-OBI reference sequences and seven genotype C sequences retrieved from GenBank labelled with their accession number. The OBI sample names on the tree correspond to the samples presented in Table 1. The vertical bar or 0.01 indicates the number of nucleotide substitutions per site. There is 100% reliability that Local References 1 to 4 and GenBank references will be grouped together. Similarly, 1A, 1C, 2C, 3A, 5A, and 5B have 100% reliability while 4A and 2B have a 99% bootstrap value.

Fig. 3 illustrates three primary groupings of the OBIs and references. All the local non-OBI and GenBank HBV genotype C reference sequences were grouped together with a bootstrap value of 100%. Samples 1A, 1C, 2C, 3A, 5A and 5B formed another group with a 100% bootstrap value, whilst 2B and 4A clustered together in a third group with a bootstrap value of 99%.

**Fig 4:**
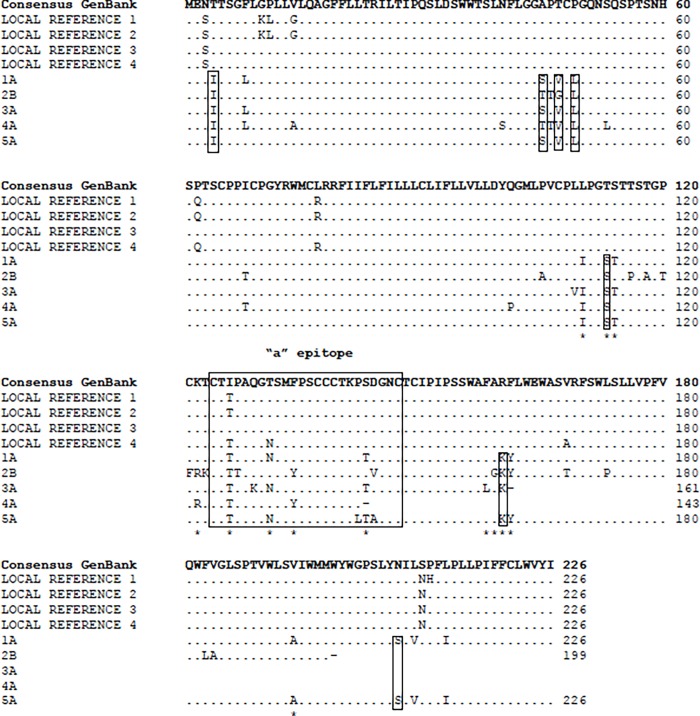
Amino acid sequence alignment of the HBV small S protein. The numbers on the right indicate the amino acid position. The common substitutions identified in this study are shown in closed boxes. The “a” epitope is shown in dashed box. Asterisks indicate known genotype- or subtype-related variability. ‘-’ Indicates stop codon. Local references were samples obtained from SEALS at POWH dating from January 2007 until March 2013 with a serological profile of HBsAg positive, anti-HBc positive and HBV DNA positive.

In order to establish the reason for this tight clustering, nucleotide and amino acid sequence alignment of the most recent samples of OBIs, non-OBI local references and GenBank consensus sequence was performed. Higher sequence variability was observed in this region in our cohort compared to reference sequences (Fig. 4). Amino acid variations at positions 110, 113, 114, 122, 126, 131, 134, 143, and 158–161 are known genotype- or subtype-related. To our knowledge however, the variations at positions 116, 118, 120, 121, 123, 127, 129, 142 and 145 may be rare mutations. Furthermore, amino acid sequences from samples 2B, 3A, and 4A carried stop codons at positions 199, 161 and 143 (within and outside the MHR) respectively, and may result in truncated S proteins (Fig. 4).

Closer examination of the nucleotide alignment of the full 681-nucleotide S gene revealed a total of eight nucleotide changes which translated into seven amino acid changes consistent in our cohort samples, as compared to reference sequences (Table 2, Fig. 4). Two of the substitutions were concurrent nucleotides, resulting in an amino acid change. Due to the overlapping nature of the S and polymerase ORFs, we were determined to investigate whether these substitutions also affect amino acid sequences in the polymerase. The first nucleotide of a polymerase codon corresponds to the third nucleotide of an S codon. Of the eight-nucleotide substitutions, only four were sense mutations and led to three amino acid changes in the polymerase ORF (Table 2).

**Table 2:** Nucleotide substitutions in the HBV S gene of OBI cohort with corresponding amino acid changes in the small S protein and polymerase.

Nucleotide Substitution	Amino Acid Substitution (Small S Protein)	Amino Acid Substitution (Polymerase)
C11T	T4I	non-sense
G133T/A	A45S/T	rtS53N/I/V
A139G and C140T/G	T47V/G	rtH55R
C146T	P49L	non-sense
A337T	T113S	rtN121I
G479A	R160K	non-sense
A620G	N207S	non-sense

To further study these substitution mutations in our cohort, we performed alignment of nucleotide sequences from patient samples collected at different time points. A summary of nucleotides at the corresponding positions is shown in Table 3.

**Table 3:** Nucleotide substitutions over time in the HBV S gene of Patients 1 and 3.

Nucleotide Position	Wild-Type Nucleotide	Patient 1		
		Day 0	Day 468	Day 1809
11	C	C	N/A	T
133	G	G	T	T
139	A	A	G	G
140	C	C	T	T
146	C	C	T	T
337	A	A	T	T
479	G	G	A	A
620	A	A	N/A	G

		Patient 3		
		Day 0	Day 495	Day 712
11	C	T	T	T
133	G	G	T	T
139	A	G	G	G
140	C	G	T	T
146	C	T	T	T
337	A	T	T	T
479	G	A	A	A
620	A	G	G	G

N/A Not Available

The earliest time-point for Patient 1, 1D (nominated 0 day), carried the wild-type nucleotide in all eight substitution sites. Serology data of this sample demonstrated HBsAg positivity (Table 1). Interestingly, the next sample collected 468 days after, 1C (nominated 468 days), had substitutions in at least six sites (we were unable to determine the nucleotides at positions 11 and 620 for this sample due to sequence quality). At a later time-point, sample 1A (nominated 1809 days) carried all eight substitutions and was also HBsAg negative.

In Patient 3 at the earliest time-point, 3C (nominated 0 day), seven of eight substitutions were observed. Nucleotide position 133 remained the same as wild-type whilst the cytosine at nucleotide position 140 was substituted by a guanine. This sample tested positive for HBsAg. In the following sample collected, 3B (nominated 495 days), the wild-type guanine in nucleotide position 133 and the guanine in nucleotide position 140 were substituted by a thymine in both positions. HBsAg testing returned negative result. The third sample collected, 3A (nominated 712 days), had substitutions in all eight sites and was HBsAg negative as well.

Subsequently, alignment of amino acid sequences obtained from GenBank was performed. When we compared GenBank OBI (n = 40) to other HBV Genotype C (n = 40), we found a significant number of amino acid substitution at position 47 (22.5% vs. 0% respectively; data not shown). There were no significant changes in all other positions.

## Discussion

Global studies of OBI show the prevalence is variable depending on the population studied, demography, and assay sensitivities used[6]. OBI infection is commonly observed in patients co-infected with non-occult HCV[14], HIV[15,16], and associated with hepatocellular carcinoma (HCC) development[17]. In Korea, HBV DNA was detected in 31/195 (15.9%) healthy Korean subjects without detectable HBsAg, anti-HCV and anti-HIV[18]. Also, a Chinese study observed HBV DNA in 15/254 (5.91%) subjects without detectable HBsAg[19]. Additionally, OBI was more prevalent in male participants (23%) as compared with female participants (8%)[18]. Although numerous studies have been conducted, the exact prevalence of OBI is still not well defined, with no study of OBI published from Australia.

Difficulties associated with the commercial HBsAg assays’ ability to detect HBsAg linked to mutations in the “a” epitope are well documented[20,21] In this study, HBsAg was tested by SEALS using Abbott Architect HBsAg Qualitative II with a sensitivity of 0.019–0.02 IU/mL. This is comparable to 17 European commercial assays with lower limits of detection 0.018 to 0.1 IU/mL using the WHO HBsAg standard[22]. Using these assays, we established that out of the 1,451 patients tested for HBsAg, anti-HBc and HBV DNA, only 0.69% (10 patients) had OBI according to the established criteria.

One of the difficulties of studying OBI is the low level of HBV DNA in serum. Using a sensitive RT-PCR protocol we were able to amplify and sequence the HBV S gene from five patients. When compared with GenBank reference sequences our cohort clustered with HBV genotype C. This genotype is common in East Asian countries and has been linked with advanced hepatic disease[23,24]. The risk of HCC for patients with genotype C was reported to be 5-fold higher than patients with genotypes A or B[25]. Genotype C is also known to be more prone to mutations in the HBV genome[26]. Previous studies have suggested mutations in the major hydrophilic region of the S gene, particularly within and surrounding the “a” epitope, are crucial for the occurrence of OBI[10,27,28]. The “a” epitope is on the MHR of the small S protein found between amino acids 124 and 147. Overall, 4/5 patients in our cohort carried mutations in the MHR that have not been associated with genotype variability. Among mutations related to immune escape, the G145R mutation has been most frequently encountered worldwide[29,30]. This mutation was absent in our cohort. However, two of our patients carried a mutation in the position adjacent to G145 (D144V/A), whilst one had a mutation at position 142 (P142I). Both have been associated with the decrease in HBsAg secretion[31]. Further amino acid residues from positions 120 to 123 were considered to be essential for the antigenicity of HBsAg[32]. One of our patients carried the C121F and T123K mutations. We also discovered premature stop codons in 3 patients, which may lead to truncated HBsAg. A previous study showed that 39% of occult samples had early S ORF termination[33]. HBsAg protein truncation may alter antigenicity leading to undetected serum HBsAg. These changes may enable the virus to persist in the liver by evading immune surveillance.

To our knowledge, previous studies have not identified any single mutation or mutations that act as markers for the occurrence of OBI. The majority of mutations reported only present in unique strains. We identified a total of seven amino acid changes outside the MHR region in the small S protein which distinguished our cohort from local and GenBank HBV reference sequences. Two of these amino acid changes, T113S and R160K, have been reported previously to be genotype/subtype-related amino acid variations. When considered in the context of the overlapping polymerase ORF, only three amino acid changes were observed, thus minimising the possibility of reduced enzymatic efficiency. Our data also suggested that these substitutions occur over time and are stable in our group of patients. This may be a consequence of reduced environmental pressures (i.e. anti-viral drugs and immune response) resulting from immune evasion. When we compared our cohort with a larger OBI cohort study performed in Korea[33], only amino acid position 47 showed significant change (22.5%). It should be noted that none of the GenBank genotype C reference sequences carried this amino acid change.

We established a prevalence of 0.69% OBI in the selected Australian cohort. In addition, we identified eight nucleotide changes which translated into seven amino acid changes in the small S protein and polymerase genes that may cause altered antigenicity and low viral replication. This may then lead to undetectable HBsAg and extremely low levels of HBV DNA in the serum. These changes may also enable the virus to persist in the liver by evading immune surveillance. Further studies of a larger cohort are required to determine whether these amino acid variations have been acquired in the process of immune escape and serve as markers of certain types of OBI.
